# Effects of incoming polygonal fault systems on subduction zone and slow slip behavior

**DOI:** 10.1126/sciadv.adu4227

**Published:** 2025-07-04

**Authors:** Maomao Wang, Philip M. Barnes, Demian Saffer, Gregory F. Moore, Haoran Ma, Ming Wang, Jinbao Su

**Affiliations:** ^1^ College of Oceanography, Hohai University, Nanjing, Jiangsu, China.; ^2^ National Institute of Water and Atmospheric Research, Wellington, New Zealand.; ^3^ University of Texas Institute for Geophysics, Austin, TX, USA.; ^4^ Department of Earth Sciences, University of Hawaii, Honolulu, HI, USA.

## Abstract

The physical properties of subduction inputs profoundly influence megathrust slip behavior. Seismic data reveal extensive polygonal fault systems (PFSs) in the input sequences of the Hikurangi Margin and Nankai Trough. The mechanical and hydrological effects of these incoming PFSs on subduction zones are potentially substantial. Here, we investigate their effects following transport into the accretionary wedge by integrating discrete-element modeling with three-dimensional seismic interpretation. We find that the typical dips of the incoming PFSs overlap with modeled dips prone to reactivation and confirm that subducting PFSs can be reactivated and gradually evolve into major thrust faults. Comparisons with electromagnetic data indicate that PFSs may provide conduits for fluid leakage along the plate interface, coincide with disrupted strata and decreased shear stress, and enhance geometric and stress heterogeneity along the megathrust. These suggest that PFSs may play a previously unrecognized role in contributing to shallow slow earthquake phenomena in subduction zones.

## Teaser

Subduction of polygonal fault systems enhance geometric and stress heterogeneity along the megathrust, promoting slow slip events.

## INTRODUCTION

**Fig. 1.**
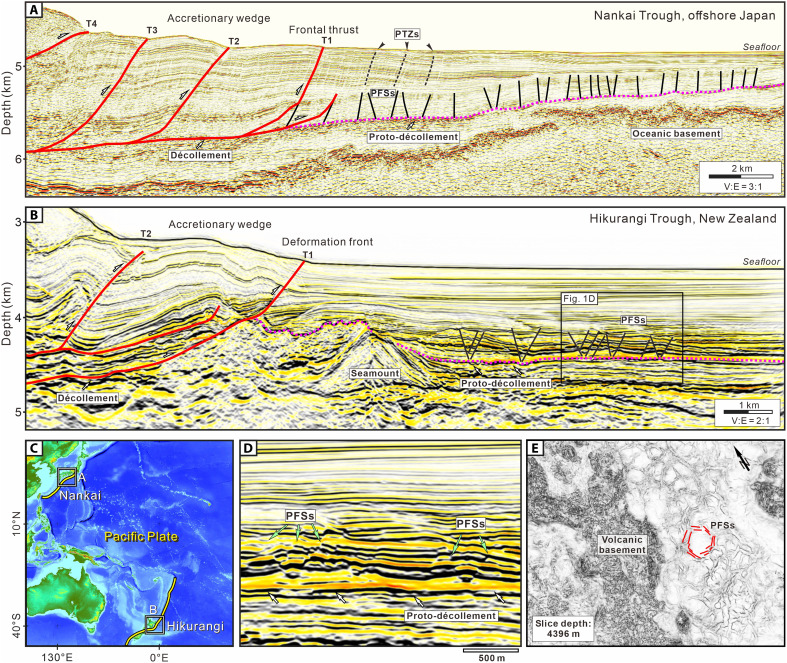
Seismic reflection profiles illustrating the frontal accretionary wedge and PFSs in Nankai Trough, Japan, and Hikurangi Trough, New Zealand. (**A**) Nankai Trough, offshore Japan. (**B**) Hikurangi Trough, New Zealand (IL 135 in NZ3D volume). (**C**) Locations of study areas. (**D**) Partial enlargement of the PFSs in (B). (**E**) Seismic coherence depth slice at 4396 m showing the PFSs in the incoming pelagic sequence. Seismic images in (D) and (E) are derived from the NZ3D seismic volume collected in the northern Hikurangi Margin (*18*).

The nature of incoming plates in subduction zones, including seafloor topography and trench sedimentation patterns, influences structural deformation within accretionary wedges and the potential for seismic slip and tsunami hazards along megathrust faults (*1*–*3*). Recently, polygonal fault systems (PFSs) have been reported in the incoming sedimentary sequences of active subduction zones, including the Nankai Trough, Japan, and the Hikurangi Trough, New Zealand (*4*–*6*) (Fig. 1). These PFSs are characterized by normal sense of displacement, polygonal structures, and close spacing, and they are bound within specific stratigraphic intervals (*7*, *8*). They form slowly during the deposition and burial history of fined-grained incoming sediments and primarily accommodate vertical contraction (*9*, *10*).

**Fig. 2.**
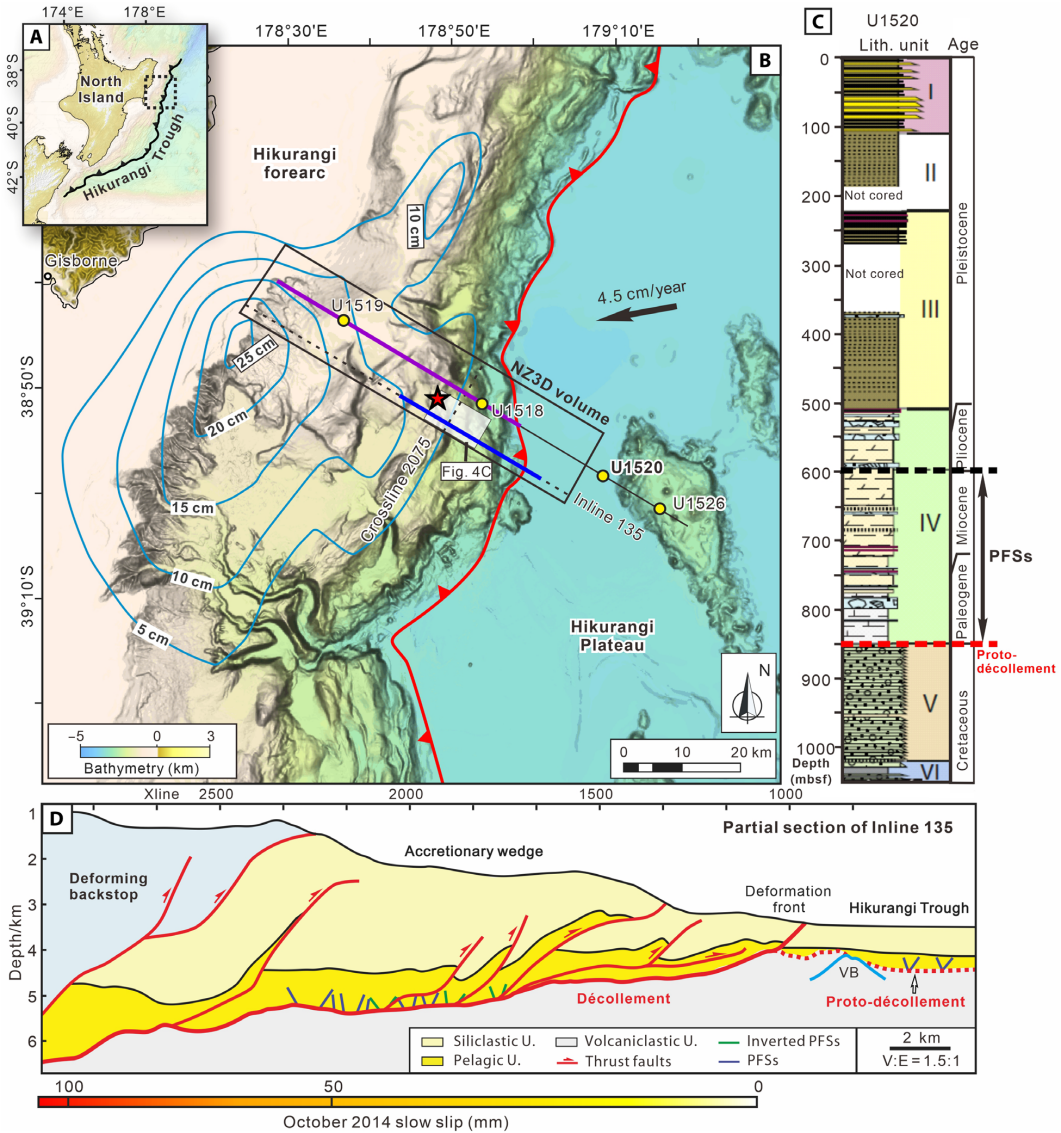
Bathymetric map showing the location of the NZ3D seismic volume at the northern Hikurangi Margin. (**A**) Regional tectonics setting. (**B**) Blue lines with labels are seismic slip contours (cm) for the September to October 2014 SSEs (*12*). White transparent rectangle demarks the location of the coherent depth slice at 5140 m shown in Fig. 4C. Red star shows the location of the 1947 tsunami earthquake. Yellow circles mark the locations of IODP Expeditions 372 and 375 drilling sites. The blue and purple thick lines show the locations of the sections in (D) and Fig. 10A, respectively. (**C**) Lithostratigraphy for IODP site U1520 (*3*, *24*). Bidirectional arrows indicate that PFSs develop primarily in calcareous, clay-rich sediments of Unit IV. (**D**) Partial section of IL 135 in NZ3D showing PFSs, seamounts, décollement, proto-décollement, and slow slip. mbsf, meters below seafloor.

Historical records and modern observations indicate that both the Hikurangi and Nankai subduction zones have generated large earthquakes, as well as recurring shallow slow slip events (SSEs), tremor, very-low-frequency earthquakes (VLFEs), and tsunamis (*11*–*14*). In particular, seafloor geodesy shows that SSEs and VLFEs at northern Hikurangi and Nankai may propagate all the way to the trench (e.g., Fig. 2), representing the shallowest and best-documented SSEs on Earth (*12*–*14*). Despite extensive observations in many active subduction zones worldwide, the physical mechanisms underlying these SSEs remain elusive (*11*, *12*). Among numerous hypotheses, the subduction of seafloor roughness and lithologic heterogeneity within the fault zone are widely invoked as primary factors (*2*).

**Fig. 3.**
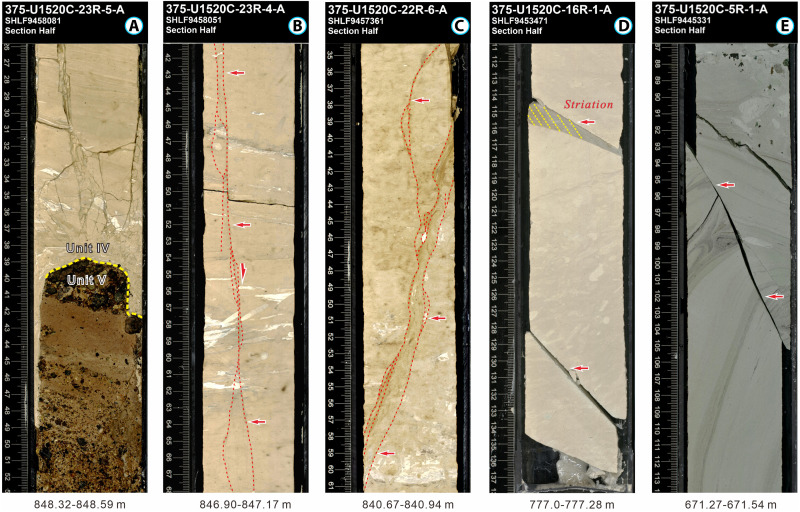
Representative core photos from IODP site U1520. (**A**) Chalk in Lithological Unit IV overlying volcaniclastic facies (Unit V). (**B**) High-angle normal faults in chalk. (**C**) Fault zone with well-developed foliation structures in chalk. (**D** and **E**) Normal faults with clear fault striations in chalk and marl, respectively.

In the Hikurangi Margin and Nankai Trough, PFSs form in pelagic carbonates and marls, and hemipelagic mudstones, respectively (*6*, *15*) (Fig. 3). These PFSs lie mostly immediately above the proto-décollement in the incoming plate and are imaged as closely spaced, conjugate normal faults with small displacements, appearing as polygonal patterns in plan view and as graben/horst structures in cross section (Fig. 1). PFSs differ from arrays of reverse faults that characterize protothrust zones (PTZs), which are characterized by parallel, closely spaced reverse faults with small displacements on seismic reflection profiles, located seaward of the primary frontal thrust (*4*, *16*). The occurrence of incoming PFSs raises important questions about their role in the structure, hydrogeology, and seismic behavior of megathrusts (*2*, *3*, *17*), including (i) Are incoming polygonal faults reactivated in the accretionary wedge during subduction? and (ii) Do they affect strain partitioning, geometric roughness, and material heterogeneity along the megathrust?

We address these fundamental questions using numerical modeling in tandem with interpretation of a recently collected 3D seismic reflection volume at the Hikurangi Margin (*18*) to investigate the kinematic and mechanical effects of incoming PFSs on the accretionary wedge and plate boundary megathrust as they are incorporated into the subduction zone. We use the discrete-element method (DEM) (*19*, *20*) to construct a series of models to investigate the fault evolution in the accretionary wedge for various polygonal fault dips. Last, we assess the implications for the structural evolution of accretionary wedges and the nature of slip along the plate interface.

### Geologic setting

At the northern Hikurangi Margin, New Zealand, the Pacific Plate is subducting westward beneath the Australian Plate at a rate of 40 to 50 mm/year (*12*, *21*) (Fig. 2). The subducting plate comprises the Hikurangi Plateau, a Cretaceous large igneous province, and its overlying volcaniclastic, pelagic, and siliciclastic sediments (*3*, *22*, *23*). In this region, the shallow plate boundary megathrust hosted two large magnitude earthquakes [*M*_w_ (moment magnitude) 7.0 to 7.2] in 1947 that produced 8- to 10-m-high tsunami along the coast of the North Island (*24*, *25*). The offshore megathrust at depths of <2 to 15 km below the seafloor also hosts well-documented recurring, short-duration (1 to 3 weeks) SSEs that recur every 1 to 2 years, as recorded by continuous GPS observations (*12*). To investigate the processes and in situ conditions that underlie these shallow SSEs, International Ocean Discovery Program (IODP) Expeditions 372 and 375 drilled several boreholes along a transect offshore of the northern Hikurangi Margin to collect samples, data, and geophysical logs and to install long-term observatories (*3*, *26*) (Fig. 2). In addition, in 2018, a 65 km–by-15 km three-dimensional (3D) seismic reflection dataset, NZ3D volume, was collected by R/V *Marcus Langseth* across the Hikurangi Margin offshore of Gisborne township (*18*) (Fig. 2B). Drilling and seismic imaging reveal that the PFSs are developed in the incoming Paleogene to Pleistocene pelagic carbonates and marls (Unit IV) beneath the Hikurangi Trough (*6*).

## RESULTS

### PFSs: From incoming Hikurangi Plateau to the SSE source region

**Fig. 4.**
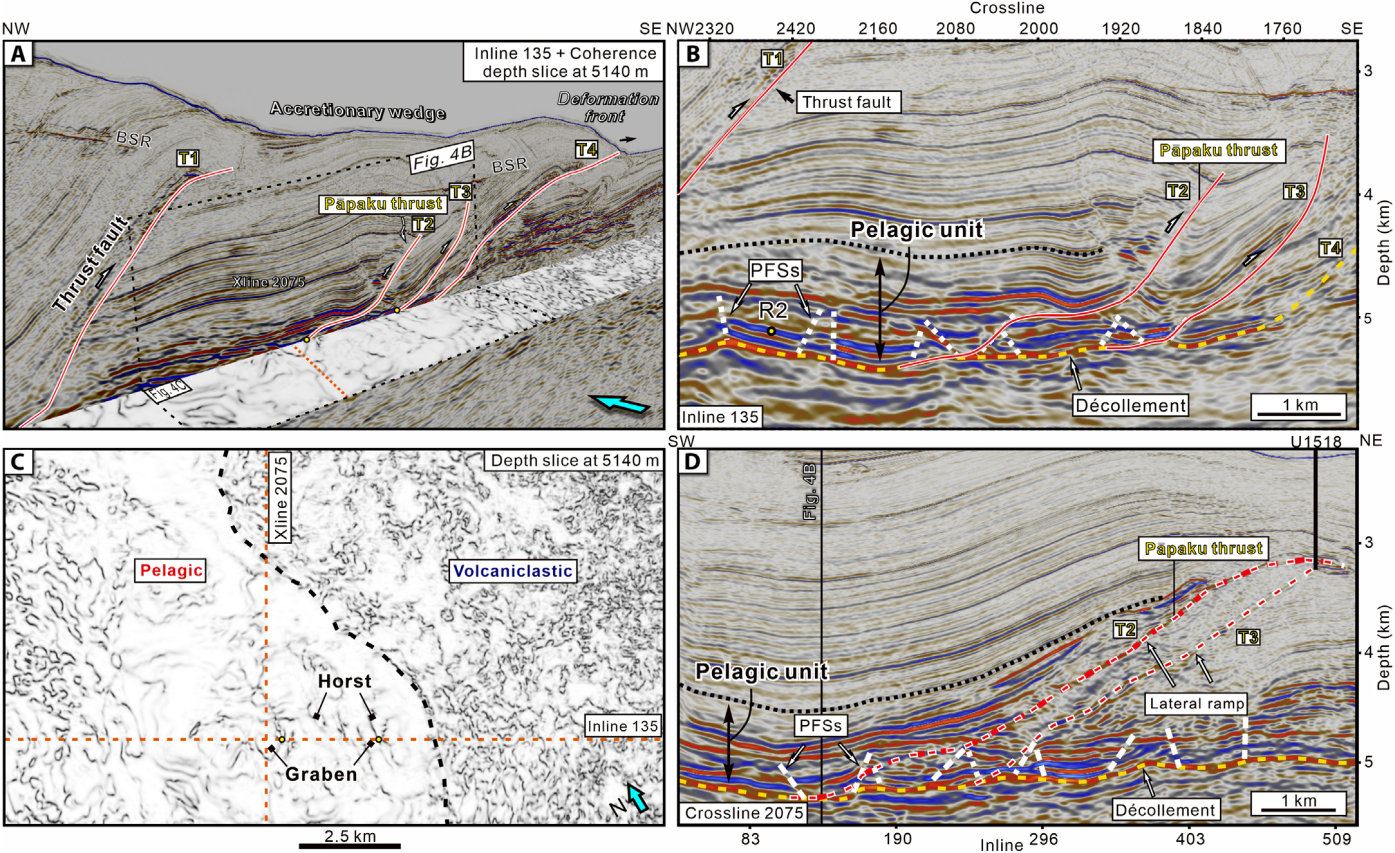
Seismic profiles and coherence depth slice from the NZ3D reflection volume showing accreted PFSs and thrust faults in the Hikurangi Margin. (**A**) Perspective view of the seismic profile (IL 135) and coherence depth slice (5140 m) looking northwest. (**B**) Enlarged panel from IL 135 showing PFSs, the southern part of the Pāpaku thrust, and other faults. (**C**) Coherence depth slice at 5140 m showing the distinct expression of PFSs in the pelagic unit within the accretionary wedge. (**D**) Crossline 2075 showing the relationship between the lateral ramp of Pāpaku thrust and PFSs through which the thrust emerges. Vertical exaggeration is 1.5:1.

**Fig. 5.**
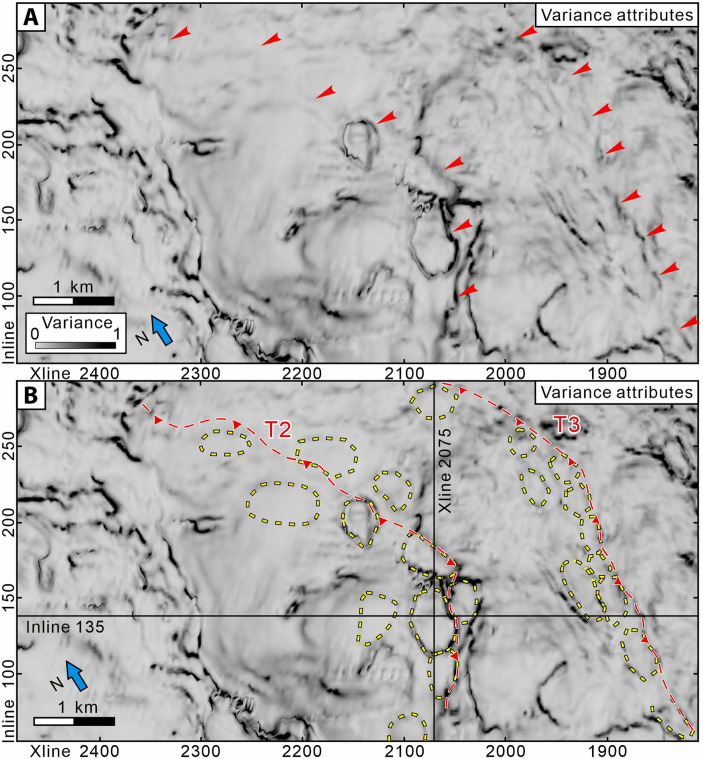
Seismic variance attribute map for the R2 reflector in Unit IV. Location of the R2 reflector is shown in Fig. 4B. (**A**) Uninterpreted variance attribute map. (**B**) Interpreted variance attribute map showing the locations of the major thrusts T2 and T3 and the PFSs.

Seismic interpretation of 178 well-imaged incoming polygonal faults from the high-resolution NZ3D seismic volume in Hikurangi Trough (*18*) indicates that the dip angles of the faults are normally distributed and range between 30° and 60°, with a mean of 48° (fig. S1). Furthermore, our structural interpretation reveals that the PFSs have been incorporated into the Hikurangi accretionary wedge in the SSE source region (Figs. 2 and 4 and fig. S2). Seismic profiles and coherence depth slices extracted from the seismic volume demonstrate the spatial relationship between the PFSs, major thrust faults, and the plate interface (Fig. 4A). From the backstop to the deformation front, a series of major thrust faults is developed (denoted as T1, T2, T3, and T4) (Fig. 4A). Among these, T2 develops northward into the Pāpaku thrust fault, a major and highly active splay fault rooted in the Hikurangi Margin plate interface that was drilled at IODP Site U1518 (Fig. 4, B and D) (*26*). A coherence slice at 5140-m depth (Fig. 4C) clearly demonstrates the polygonal structure in the accreted pelagic unit immediately above the plate interface. In addition, we also picked the R2 reflector within Unit IV (location shown in Fig. 4B), which is affected by the PFSs, and generated a 3D horizon map displaying the variance attribute. This map shows a strong correlation between the locations of PFSs and major thrusts T2 and T3 formed in the accretionary wedge (Fig. 5).

As in the subduction input sequences, in cross section, the accreted PFSs are layer bound and confined to the pelagic unit (Fig. 4, B and D). The thrust ramp of T2 extends upward through the PFSs, incorporating and reactivating the inherited structure. Along strike, we interpreted a total of 65 seismic Inlines (ILs) and we illustrate the position of T2 on XL (crossline) 2075 (Fig. 4D). The results show that a lateral ramp on the incipient Pāpaku thrust in the south on IL 135, hosted in the pelagic section, gradually evolves along-strike to accommodate multikilometer displacement in the northeast. Thus, IL 135 captures the incipient stages of major thrust development. Our 3D seismic interpretation thus confirms that accreting PFSs lie in proximity to the megathrust interface and can be reactivated in the early stages of major thrust fault development in the accretionary wedge.

### Numerical modeling results

**Fig. 6.**
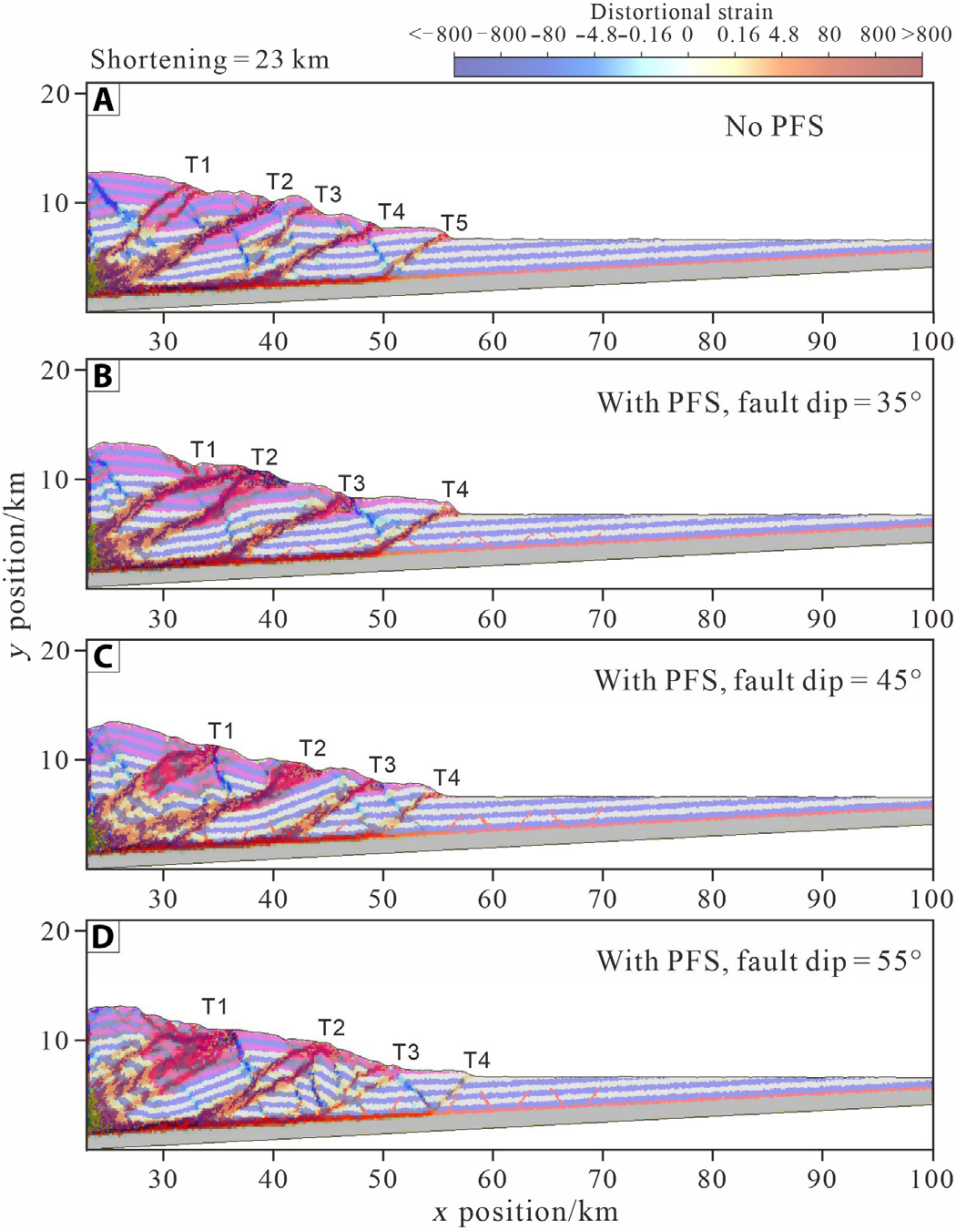
Comparison of structural deformation without and with polygonal faults in accretionary wedges based on DEM simulation. (**A**) Without polygonal faults and (**B** to **D**) with polygonal faults at dip angles of θ = 35°, 45°, and 55°, respectively. The red + blue and white + blue areas represent trench fill and pelagic sediment units, respectively. T1 to T6 represent the thrust faults generated sequentially during the deformation process. The deformation strain field is superimposed on the colored layers.

To further investigate the kinematic and mechanical effects of the incoming PFSs, we use discrete-element modeling because it can reproduce Coulomb failure and form cracks or brittle/opening-model failures and generate faults under applied stress conditions (*19*, *20*). This technique allows us to quantitatively analyze the evolution of strain and stress to reveal fault activity and the mechanical evolution of an accretionary wedge. We conducted comparative experiments to investigate the effect of fault dips of PFSs on kinematic and mechanical evolution in the wedge (Figs. 5, A to D, and 6, A to D, and figs. S3 and S4). A model without PFSs served as the reference model (Fig. 6A), whereas in the models containing PFSs, the fault dip angles were set to 35°, 45°, 55°, 65°, and 75° (Fig. 6 and movies S1 to S4). In the incoming Hikurangi plate, the average width of grabens ranges from 1 to 1.2 km, whereas that of horsts ranges from 0.4 to 0.5 km. The lateral extension of a single PFS is only 15 to 33% of that of fault spacing (mean 3.7 km) of major thrusts in Hikurangi wedge. This implies that the width of an individual PFS does not influence the formation and development of thrust faults; instead, the combination of multiple PFSs to form wider grabens plays a dominant role. Therefore, we designed a set of models with narrower and wider configurations to investigate the impact of multiple PFS spacings on the thrusting process. In the above experimental groups (Model I), the spacing between graben and horst in PFSs was 4.2 and 2.2 km, respectively, and for comparison, we designed another set of experiments (Model II) (movies S5 and S6) where the corresponding widths were reduced to 3.2 and 1.2 km, respectively. Moreover, geophysical surveys reveal many seamounts are subducting along the northern Hikurangi Margin. Thus, it is essential to investigate how the subduction of seamount coupled with PFSs affects the structural deformation of the accretionary wedge and thrust development. Therefore, we conducted a 2D DEM experiment incorporating both a seamount and PFSs, where the PFS fault dip was set at 55°, and the seamount set as 15 km in width and 1 km in height (fig. S3C).

In models with fault dips of 35° and 45°, as strain propagates along the detachment into the PFS region, fore-thrusts initially develop along preexisting, land-dipping faults, such as T3 in the 35° model and T2 in the 45° model (Fig. 6 and fig. S4). Models with fault dips of 55°, 65°, and 75° predominantly favor back thrusting, leading to the creation of pop-up structures. In the model with fault dip of 55°, structural strain slips along the preexisting, offshore-dipping PFS fault plane, forming back-thrusts. In contrast, in the 65° and 75° models, strains do not directly propagate along the two fault planes in PFSs but instead generate pop-up structures between them. This suggests that high-dip input PFSs do not readily activate preexisting faults but rather contribute to constraining the location of younger formed thrusts (movies S1 to S4).

**Fig. 7.**
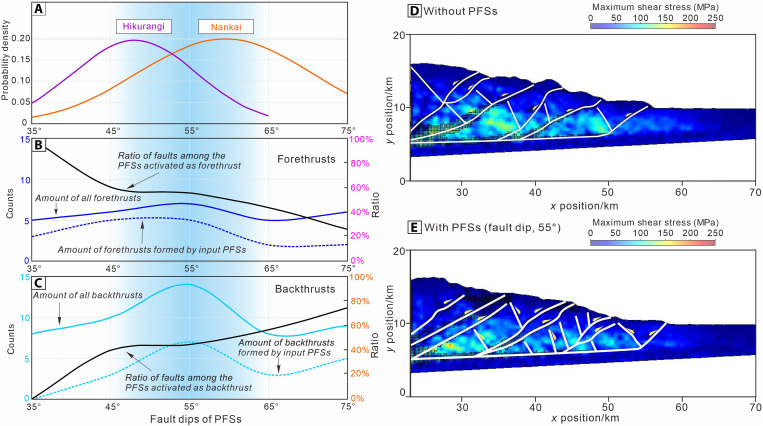
Statistical data for modeled fault structures and maximum shear stresses field in the accretionary wedge. (**A**) Probability density function of the subducting PFS fault dips calculated from seismic reflection data in Hikurangi and Nankai margins. The blue transparent regions of (A) to (C) represent the dip range (45° to 65°) that is most favorable for the reactivation of PFSs. (**B** and **C**) Quantity and proportion of fore-thrusts and back-thrusts faults formed in the DEM Model I under various PFS fault dips. (**D** and **E**) Distribution of maximum shear stresses within the wedges in the smooth reference model and the PFS-bearing model (fault dip, θ = 55°). Model shortening is 23 km.

In the reference model, thrust faults form successively in a breaking-forward manner, with the growth of the young fault occurring after the cessation of the preceding fault (Fig. 6A and fig. S4). In the PFS-bearing model with fault dip of 45°, the fore-thrusts T2 to T6 all develop on the preexisting PFS, indicating a strong correlation (83%) to the preexisting PFS (Figs. 6C and 7, and fig. S4). The statistical results indicate that, within the range of fault dip angles between 45° and 65° in the incoming PFSs, the modeled accretionary wedge forms the greatest number of fore-thrust and back-thrust faults. As fault dip increases from 55° to 75°, the correlation between fore-thrust development and input PFS decreases from 71 to 33% (Fig. 7). In the model with a 35° fault dip, fore-thrusts account for 100% of PFS activations. As PFS fault dip increases from 45° to 75°, the proportion of fore-thrusts declines from 60 to 25%, whereas back-thrusts increase from 40 to 75%. This indicates that PFSs with moderate fault dips (35° to 55°) are more likely to be activated as fore-thrusts, absorbing large displacements, whereas steeper faults (≥65°) tend to reactivate as back-thrusts and are less favorable for activation (Fig. 7, B and C).

**Fig. 8.**
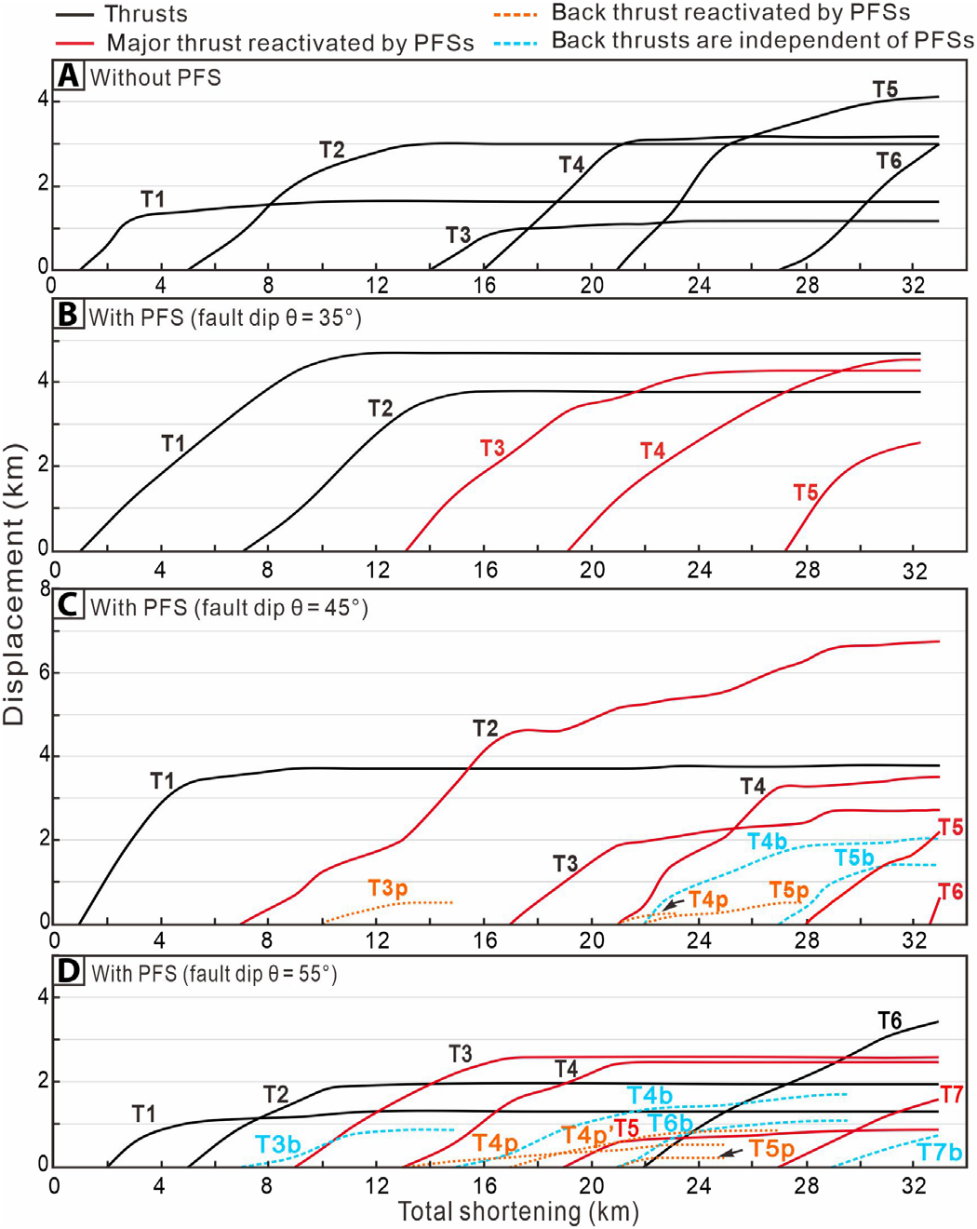
Graphs showing a comparison of the fault displacement versus tectonic shortening for DEM experiment groups. Statistics of fault activity in the accretionary wedge without (**A**) and with (**B** to **D**) PFSs (fault dips = 35°, 45°, and 55°) as a function of tectonic shortening. T1 to T6 represent thrust faults, with the red lines indicating reactivated inherited PFS segments. T4b and T5b represent back-thrusts corresponding to the main T4 and T5 thrusts. T3p, T4p, and T5p denote activated inherited PFS segments located immediately above the detachment.

Figure 8 illustrates the modeled displacement profiles of thrusts T1 to T6 with increasing total shortening, for model comparisons with and without PFSs. The maximum thrust displacement in the reference model reaches ~4.1 km for T5 (Fig. 8A). In the PFS-bearing model with fault dip of 45°, T1 begins to form as the subduction of input particles progressively creates a wedge-shaped thrust structure (Fig. 6C), similar to the reference model. As T1 reaches ~3.8-km displacement, T2 becomes active and continues to absorb a substantial amount of displacement during the shortening phase of 7 to 32 km. T2 reaches its maximum displacement of ~6.8 km, far exceeding that of T2 in the reference model (maximum displacement of 3 km). As shown on the displacement-shortening graph (θ = 45°) (Fig. 8C), the fore-thrusts T2 to T6 all develop on the preexisting PFS, indicating a strong correlation to the preexisting PFS. Eventually, the model develops five back-thrusts: T3p, T4p, and T5p, near the bottom of accretionary wedge, are developed by the input PFS, whereas T4b and T5b are independent of the input PFS. However, the displacement of single T3p, T4p, and T5p does not exceed 1 km, reflecting weak connection to the seaward-dipping faults within the PFS (Fig. 8C and fig. S4C). In the model with PFS at θ = 55°, the fore-thrusts also exhibit a forward-breaking characteristic. Seven fore-thrusts are produced with a maximum displacement under 2 km and an average displacement between 0.5 and 1.5 km (Fig. 8D). Because of the development of back-thrusts, the total displacement accommodated by individual fore-thrusts within the accretionary wedge is limited to no more than 3 km (excluding T6), which is less than that observed in the model with θ = 45° (Fig. 8C and fig. S4D).The DEM simulations reveal that the fault dip angle range (40° to 60°, averaging 48°) of the PFSs in the input sequence of the Hikurangi Margin falls within the range (45° to 65°) favorable for accretionary wedge fault reactivation. This explains the utilization of such structures at the earliest stages of thrust development observed in the 3D seismic volume (Fig. 4). We surmise that, with increasing displacement accruing on growing thrusts (along strike and through time), the irregular inherited PFS geometry becomes progressively smeared out as these structures are incorporated in the wider thrust damage zone. We compared the differences in the stress field at the frontal region of the accretionary wedge with and without PFS accretion. Our numerical modeling results show that the frontal wedge of the PFS-bearing model (at shortening = 23 km, fault dip = 55°) is divided into smaller, low-stress, scattered bodies than in the reference model with a potentially smoother interface (Fig. 7, D and E).

We investigated the impact of two different graben spacings in the input PFS on the structural evolution of accretionary wedges. Two models show a recurring pattern marked by gradual internal shortening and rapid forward propagation (fig. S5 and movies S5 and S6). Notably, in Model II experiments, the distance from the backstop to the deformation front typically exceeds 1.3 to 1.6 times that of Model I. That is, Model II experiments produce a wider accretionary wedge. The deformation cycle in Model II is shorter compared to Model I, roughly ~33 to 50% of the latter duration (fig. S5). Analysis of mean wedge surface slopes reveals that all simulated accretionary wedges reach a critical state when model shortening reaches 22 km. Most mean wedge surface slopes stabilize around 15° ± 1°, except for Model II-45°, which exhibits a slightly lower slope of 12° to 13°.

Models of the coupling between seamounts and PFSs suggest that, during the subduction process, PFSs lying above volcanic relief can be reactivated to form fore-thrusts or back-thrusts. PFSs in the leading flank of the seamount are gradually transported to the top of the seamount and can also be reactivated (fig. S6). At the trailing flank of the seamount, however, the strata are protected and PFSs are not activated. As subduction continues, the detachment continues to propagate forward past the seamount, activating the input PFSs to form the frontal thrust faults (at shortening = 29 km) (fig. S6 and movie S7).

## DISCUSSION

### Influences of PFS accretion on structure, fluid flow, and SSEs

**Fig. 9.**
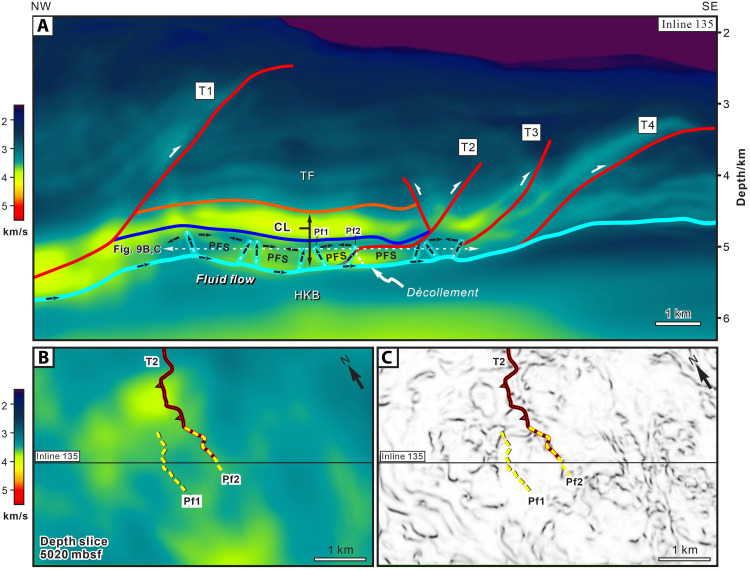
*P*-wave velocity and fault-stratigraphic architecture of IL 135 in the NZ3D demonstrates the effect on fluid flow along the décollement and PFSs in the accretionary wedge. (**A**) The locations of major thrusts, horizons, décollement, and PFSs are derived from the interpretation of the corresponding seismic reflection profile. (**B** and **C**) Vp and coherence slice at depth 5020 mbsf centered on IL 135 in NZ3D volume showing the heterogeneity of velocity and locations of PFSs (Pf1 and Pf2) and thrust fault (T2). Abbreviations: TF, hemipelagic turbidites; CL, calcareous pelagic sediments; HKB, the volcaniclastics of the Cretaceous Hikurangi Plateau.

The *P*-wave velocity model obtained from 3D full-waveform inversion (FWI) at the north Hikurangi Margin (*18*) combined with the structural interpretation of IL 135 reveals the influence of inherited PFSs on the velocity structure in the lower accretionary wedge, directly above the plate interface (Fig. 9 and fig. S7). In contrast to the incoming sequence, the accreted calcareous pelagic unit consists of two velocity zones: an upper zone of continuous reflectors with high velocities, ranging from ~3.8 to 4.0 km/s, and a lower zone containing PFSs and having lower velocities ranging from ~3.4 to 3.5 km/s (Fig. 7A). The lower zone exhibits a heterogeneous velocity structure, with zones or “halos” of higher *P*-wave Velocity (Vp) coincident with polygonal faults (Fig. 9, B and C). This correlation suggests that PFSs may provide permeable conduits for fluid flow, leading to enhanced consolidation of the surrounding sediment.

**Fig. 10.**
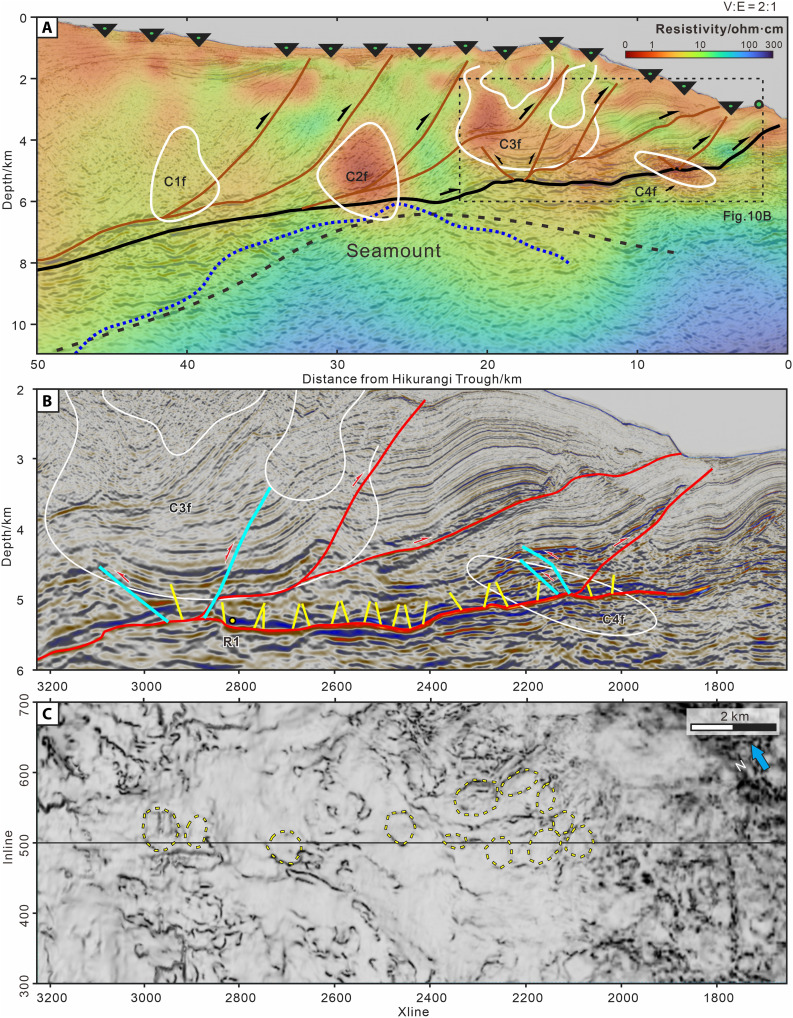
Comparison of the HT-RESIST resistivity model with NZ3D seismic data. (**A**) Colocated seismic profile IL 500 from NZ3D (*18*) overlaid on the resistivity model (*27*). C1f, C2f, C3f, and C4f are four prominent conductors identified by (*27*). The black and blue dashed lines represent magnetic basement (*42*) and seismic reflection seamount (*3*), respectively. (**B**) Enlarged seismic reflection profile corresponding to the C3f and C4f region. (**C**) Seismic variance attribute map for the R1 reflection layer in Unit IV. Location for R1 is shown as a yellow dot in (B).

Comparing the HT-RESIST electromagnetic profile (*27*) with NZ3D seismic data reveals the effects of incoming PFSs on fluid transport during subduction (Fig. 10 and fig. S8). The controlled source electromagnetic method (CSEM) effectively image fluid-saturated sediments and fractured rock by detecting conductive phases (*28*, *29*). However, it is important to recognize that the spatial resolution of the CSEM models is quite limited, especially in the vertical dimension. In the HT-RESIST resistivity profile, four broad low-resistivity zones (C1f, C2f, C3f, and C4f) were identified above the plate-boundary interface. Among them, C2f is located on the top of a seamount, which is interpreted as fluids transferred to upper plate through tensional failure fracture during seamount subducting. However, in the C3f, and C4f regions (Fig. 10A), we have identified both plan-view and cross-sectional evidences of PFSs. Notably, in the C3f and C4f regions, accreted PFSs have been reactivated as thrust faults, with their distribution aligning well with the low-resistivity zones (Fig. 10C). We identify a region of relatively high resistivity at the trailing edge of a seamount, between C2f and C3f. This region corresponds to the strata-protected zone at the seamount’s trailing edge in the seamount-PFS coupling DEM simulation (fig. S6). We interpret this high-resistivity region to result from the fact that the PFSs here are not activated and therefore do not provide a conduit for upward migration of fluids. Considering both the seismic velocity and resistivity data, we thus suggest that the reactivation of polygonal faults may provide pathways for fluid transport from the lower wedge as well as transient leakage from underlying fluid-rich subducting volcaniclastics (*30*). Polygonal faults opened under reactivation may both provide a mechanism for transport of fluids upward through the accretionary prism, which may be linked to tremor and microseismicity, and contribute to heterogeneity in fluid pressure and stress state at and immediately above the plate interface (*6*).

We have demonstrated above that the typical dips of the north Hikurangi incoming PFS (40° to 60°, mean 48°) and Nankai incoming PFSs (45° to 75°, mean 59°) overlap the range of modeled dips (45° to 65°) that are prone to reactivation as fore-thrust and back-thrust faults within the accretionary wedge (Fig. 7). The observed differences in PFS fault dip angles between these two subduction zones may reflect variations in sediment lithology and water content. In the Hikurangi Margin, PFSs primarily develop in pelagic calcareous rocks (including chalk and marl), whereas in the Nankai margin, they occur in hemipelagic mudstones. Generally, calcareous rocks tend to have higher internal friction angles than mudstones (*31*). One possible explanation for the difference in PFSs dip is that, in the Hikurangi Trough, pressure solution processes have led to the formation of numerous stylolites in Unit IV carbonate rocks, which create localized weakness zones and reduce the overall mechanical strength of the sediments (*32*). However, because the fault dip angles of PFSs in both regions fall within the activation-prone range of 45° to 65°, this difference does not notably affect the probability of thrust fault formation during accretion. The average lateral extent of PFSs within the input sequence is ~1 km, whereas the major thrust faults (T1, T2, and T3) within the accretionary wedge have an average lateral spacing of 3.7 km (Fig. 4). Specifically, the average distance between T1 and T2 is 6.7 km and between T2 and T3 is 3 km. This indicates that the lateral extent of PFSs is about 15 to 33% of that of the major thrust faults. In addition, the lateral length of individual PFSs is small and negligible compared to the major thrusts. Therefore, under compressive force, thrust faults will preferentially form in those preexisting PFSs that are parallel to their strike.

Numerical and physical modeling show that, in comparison with relatively smooth subduction interfaces, rough and rheologically heterogeneous subduction interfaces exhibit lower fault strength (*33*–*36*) and interseismic coupling and generate smaller rupture areas and shorter-duration SSEs with smaller mean displacements (*37*, *38*). Such heterogeneity in subduction interface physical properties has been attributed to seamount subduction and spatially varying lithologies (*3*, *39*–*46*), patchiness in velocity structure and pore pressure, and occurrence of fluid-rich subducting volcaniclastics (*16*, *23*). In addition, our DEM simulations, analysis of the NZ3D seismic data, and comparison with HT-RESIST electromagnetic model reveal that subducting PFSs also affect the structural evolution of the accretionary wedge, imparting heterogeneity in stress, thrust fault geometry, and possibly fluid pressure. Furthermore, we infer that, if parts of the shallow plate interface propagate through the lower PFS, the complex 3D structure of the inherited PFS may impart interface fault zone geometric irregularity at scales of 10^1^ to 10^3^ m. Thus, we conclude that PFS accretion and reactivation may contribute to variations in interface fault slip behavior and potentially to SSE processes.

Extensive studies have shown that well-characterized shallow SSEs occur in the Nankai, Hikurangi, Costa Rica, and North Japan Trench (*2*). To date, PFSs have been reported only at Hikurangi and Nankai (*6*, *15*). At present, therefore we can conclude only that PFSs have played a role in the occurrences of shallow SSEs at the Hikurangi and Nankai subduction margins. We believe, however, that future high-resolution 3D seismic imaging will be instrumental in confirming the presence of PFSs in the incoming plates of other subduction zones.

## MATERIALS AND METHODS

### Discrete-element method

The DEM system consists of a series of elastic-frictional particles, where each particle moves independently, generating normal and shear forces in contact with neighboring particles (*19*, *20*). In the DEM, bonds are usually formed between particles to simulate the cohesion of natural rocks, which can deform under tensile and shear forces, and break when the forces exceed the tensile or shear strength of the particles. Thus, the DEM can effectively simulate the structural evolution and fault activity in contractional fold-and-thrust belts (*20*, *44*, *47*). The DEM system comprises a series of elastic-frictional particles, with each particle moving independently and generating normal (*f*_n_) and shear forces (*f*_s_) through contact with neighboring particles. The normal and shear forces during particle interactions are calculated as follows

$$f_{n}=k_{n}\delta_{n}$$ (1)

$$f_{s}=k_{s}\delta_{s}$$ (2)

where *k*_n_ denotes the normal interparticle stiffness and δ_n_ represents the overlap between particles. In DEM, bonds are typically formed between particles to model the cohesion of natural rocks. These bonds can deform under tensile and shear forces and will break when the forces exceed the tensile or shear strength of the particles. Particles can slide past each other, and faults begin to develop and grow when the following conditions are met

$$f_{s}\leq \mu_{p}f_{n}$$ (3)

where μ signifies the friction on the particle surfaces. By calculating the resultant forces on each particle, which primarily include gravity, interparticle contact forces, and external forces from the boundary wall, the displacement and velocity of each particle can be determined.

### Experimental design

The numerical code used for DEM modeling in two dimensions (2D), VBOX or Virtual Sandbox (*47*), is accessible publicly and available on the website (https://geovbox.com/en/). This code was written in C language, and the parallel design was completed using OpenMP (*47*). The code was modified from RICEBAL, developed at Rice University by J. Morgan (*20*), which was modified from the original open-source code TRUBAL, developed by P. Cundall and O. Strack (*19*). The processing scripts used in VBOX were also developed and shared by J. Morgan (*20*), which used GMT (*48*).

In this study, we conducted 2D DEM simulations based on two primary considerations. First, model simplification: Using 2D DEM allows us to focus on key factors influencing fault reactivation, particularly fault dip angle and spacing. Considering the 3D complexity of the PFSs, we selected a cross section through the center of the PFSs, aligned parallel to the compression direction, as the basis for our initial 2D DEM model. Under these conditions, the cross section appears as several groups of oppositely dipping faults, separated from one another, resembling the structure shown in Fig. 4B. This setup allows us to better capture the key structural characteristics of the PFSs. Second, computational cost and time: The current version of VBOX supports only 2D simulations, and our existing model involves ~150,000 particles per simulation. A 3D model would require about 1.5 million to 3 million particles, notably increasing computational cost and time. However, in 3D simulations, we can anticipate that additional complexities will emerge.

The models are set up based on the structural profiles, mechanical stratigraphy, and décollement dip angles at the Hikurangi and Nankai subduction margins in New Zealand and Japan, respectively (*3*, *6*, *18*). These two margins share several key similarities—and exhibit characteristics common to accretionary subduction zones more generally—that carry broader implications. We set up stratified geomechanical models that contain (i) low cohesion trench-wedge representing sediments, (ii) a constant-thickness, high cohesion calcareous sequence, (iii) a detachment, and (iv) oceanic crustal basement (fig. S3). Bulk mechanical properties assigned to the stratified units and faults are listed in table S1.

The model’s width was set to 80 km, filled with ~150,000 mixed particles of 60- and 80-m radii (fig. S3). DEM focuses on the microscopic processes of the medium. In DEM, the geological body is simplified into an assemblage of discoid elements. The diameter of the particles affects the structural scale of the simulations. If the diameter of the particles decreases, the number of particles and the calculation time will increase. There is a balance between accuracy and calculation speed. We refer to a previous study (*20*), and the diameter of the particles (60 and 80 m) is consistent with their findings. In DEM simulations, the particles in the discrete element are not intended to represent the actual particles in the rock (grains) but serve only as computational units (elements) in the numerical simulation, whereas DEM for geological applications typically uses larger particle sizes and fewer particles to model structures at a larger spatial scale that are applicable to common geological problems. The simulation results of models with different particle sizes indicate that they have a minimal impact on the overall morphology of the structure in the model (*49*). A comprehensive, detailed description of the theory behind this modeling approach and the methods used to justify the parameter values for DEM has been provided in (*20*, *47*).

Our analysis of the Hikurangi accretionary wedge using NZ3D data reveals décollement dip angles ranging from 1.9° to 4.9° (mean 3°). Comparable seismic profiles from the Nankai margin (*50*) show similar shallow dips of 0.5° to 4.0° (mean 2°). These findings indicate that both margins feature low-angle décollements (≤3°) in their frontal wedge regions. Accordingly, we implemented a model configuration with (i) a 3° basal dip, (ii) 8-km left-side height, and (iii) 6-km combined thickness for trench-wedge and carbonate sequences (fig. S3). In the PFS-bearing model, the fault dips were set to 35°, 45°, 55°, 65°, and 75°, respectively (fig. S3). We established two model groups with different fault spacing, labeled as Models I and II. In Model I, the spacing between graben and horst in the PFS is set as 4.2 and 2.2 km, respectively, whereas in Model II, these spacings are adjusted to 3.2 and 1.2 km, respectively. In terms of model setup, it is technically challenging to configure smaller-scale grabens and horsts. This is because smaller-spaced, lower dip faults will cause the upper segments of the faults to cross each other. A previous study has indicated that the spacing of thrust faults is primarily influenced by the cohesion within the wedge (*20*); thus, the effect of more closely spaced PFSs can be considered negligible. The width of the grabens/horsts in our model, as well as the ratio of graben/thrust fault spacing, closely approximates that observed in structural cross sections from Hikurangi and Nankai margins. In addition, we also designed a 2D DEM experiment to include both a seamount and PFSs. In this study, the seamount—composed of basalt—shares the same composition as the oceanic crustal basement. To model its subduction, we introduced a bulge of specific width and height along the top of the basement. The configuration of the seamount is primarily based on its size constrained by seismic line 05CM-04 (*3*). The seamount was set with a width of 15 km and height of 1 km, and the fault dip of the PFSs was set to 55° (fig. S3).

DEM simulations must maintain quasistatic conditions to properly model tectonic deformation processes at geological timescales. Previous calibration tests using biaxial compression (a standard rock mechanics testing configuration) demonstrate that specimen peak stress increases sharply at compression rates ≥ 2 m/s while remaining relatively stable at rates ≤ 2 m/s (*20*, *47*). This confirms that rates ≤ 2 m/s produce quasistatic conditions where the compression rate has minimal impact on deformation and damage processes. Thus, we set the compression rate to 2 m/s, which means that the simulation is ensured to be a quasistatic process, whereas the time and cost consumed by the calculation are also acceptable (*20*, *47*).

In this study, we adopt the concept of distortional strain as defined by Morgan (*20*) to illustrate strain and shear sense during structural deformation. In the output images from DEM, distortional shear is represented by color intensity, with red indicating right-sense shear and blue indicating left-sense shear. Fault displacement is measured from specific marker layers within the model. The output animations of DEM simulation are provided in the Supplementary Materials (movies S1 to S7).

### NZ3D seismic data

Details of the NZ3D seismic reflection data acquisition, processing, and availability can be found in (*18*) and (*51*). In addition, details of the seismic data processing, including derivation and accuracy of the velocity field, can be found in a seismic data processing report (*52*).

## References

[1] K. Wang, S. L. Bilek, Do subducting seamounts generate or stop large earthquakes? Geology 39, 819–822 (2011).

[2] D. M. Saffer, L. M. Wallace, The frictional, hydrologic, metamorphic and thermal habitat of shallow slow earthquakes. Nat. Geosci. 8, 594–600 (2015).

[3] P. M. Barnes, L. M. Wallace, D. M. Saffer, R. E. Bell, M. B. Underwood, A. Fagereng, F. Meneghini, H. M. Savage, H. S. Rabinowitz, J. K. Morgan, H. Kitajima, S. Kutterolf, Y. Hashimoto, C. H. E. De Oliveira, A. Noda, M. P. Crundwell, C. L. Shepherd, A. D. Woodhouse, R. N. Harris, M. Wang, S. Henrys, D. H. N. Barker, K. E. Petronotis, S. M. Bourlange, M. B. Clennell, A. E. Cook, B. E. Dugan, J. Elger, P. M. Fulton, D. Gamboa, A. Greve, S. Han, A. Hüpers, M. J. Ikari, Y. Ito, G. Y. Kim, H. Koge, H. Lee, X. Li, M. Luo, P. R. Malie, G. F. Moore, J. J. Mountjoy, D. D. McNamara, M. Paganoni, E. J. Screaton, U. Shankar, S. Shreedharan, E. A. Solomon, X. Wang, H.-Y. Wu, I. A. Pecher, IODP Expedition 372 Scientists, Slow slip source characterized by lithological and geometric heterogeneity. Sci. Adv. 6, eaay3314 (2020). 32232148 10.1126/sciadv.aay3314 PMC7096157

[4] H. L. Tilley, G. F. Moore, M. Yamashita, S. Kodaira, Along-strike variations in protothrust zone characteristics at the Nankai Trough subduction margin. Geosphere 17, 389–408 (2021).

[5] A. D. McArthur, D. E. Tek, Controls on the origin and evolution of deep-ocean trench-axial channels. Geology 49, 883–888 (2021).

[6] M. Wang, P. M. Barnes, J. K. Morgan, R. E. Bell, G. F. Moore, M. Wang, A. Fagereng, H. Savage, D. Gamboa, R. N. Harris, S. Henrys, J. Mountjoy, A. M. Tréhu, D. Saffer, L. Wallace, K. Petronotis, Compactive deformation of incoming calcareous pelagic sediments, northern Hikurangi subduction margin, New Zealand: Implications for subduction processes. Earth Planet. Sci. Lett. 605, 118022 (2023).

[7] J. Cartwright, Diagenetically induced shear failure of fine-grained sediments and the development of polygonal fault systems. Mar. Pet. Geol. 28, 1593–1610 (2011).

[8] J. J. King, J. A. Cartwright, Ultra-slow throw rates of polygonal fault systems. Geology 48, 473–477 (2020).

[9] J. Watterson, J. Walsh, A. Nicol, P. A. R. Nell, P. G. Bretan, Geometry and origin of a polygonal fault system. J. Geol. Soc. London 157, 151–162 (2000).

[10] B. J. Tewksbury, J. P. Hogan, S. A. Kattenhorn, C. J. Mehrtens, E. A. Tarabees, Polygonal faults in chalk: Insights from extensive exposures of the Khoman Formation, Western Desert, Egypt. Geology 42, 479–482 (2014).

[11] S. Y. Schwartz, J. M. Rokosky, Slow slip events and seismic tremor at circum-Pacific subduction zones. Rev. Geophys. 45, RG3004 (2007).

[12] L. M. Wallace, Slow slip events in New Zealand. Annu. Rev. Earth Planet. Sci. 48, 175–203 (2020).

[13] E. Araki, D. M. Saffer, A. J. Kopf, L. M. Wallace, T. Kimura, Y. Machida, S. Ide, E. Davis, IODP Expedition 365 shipboard scientists, Recurring and triggered slow-slip events near the trench at the Nankai Trough subduction megathrust. Science 356, 1157–1160 (2017). 28619941 10.1126/science.aan3120

[14] M. Nakano, T. Hori, E. Araki, S. Kodaira, S. Ide, Shallow very-low-frequency earthquakes accompany slow slip events in the Nankai subduction zone. Nat. Commun. 9, 984 (2018). 29540688 10.1038/s41467-018-03431-5 PMC5852141

[15] A. S. Heffernan, J. C. Moore, N. L. Bangs, G. F. Moore, T. H. Shipley, Initial deformation in a subduction thrust system: Polygonal normal faulting in the incoming sedimentary sequence of the Nankai subduction zone, southwestern Japan. Geol. Soc. Lond. Mem. 29, 143–148 (2004).

[16] P. M. Barnes, F. C. Ghisetti, S. Ellis, J. K. Morgan, The role of proto-thrusts in frontal accretion and accommodation of plate convergence, Hikurangi subduction margin, New Zealand. Geosphere 14, 440–468 (2018).

[17] J. D. Kirkpatrick, J. H. Edwards, A. Verdecchia, J. W. Kluesner, R. M. Harrington, E. A. Silver, Subduction megathrust heterogeneity characterized from 3D seismic data. Nat. Geosci. 13, 369–374 (2020).

[18] N. L. Bangs, J. K. Morgan, R. E. Bell, S. Han, R. Arai, S. Kodaira, A. C. Gase, X. Wu, R. Davy, L. Frahm, H. L. Tilley, D. H. N. Barker, J. H. Edwards, H. J. Tobin, T. J. Reston, S. A. Henrys, G. F. Moore, D. Bassett, R. Kellett, V. Stucker, B. Fry, Slow slip along the Hikurangi margin linked to fluid-rich sediments trailing subducting seamounts. Nat. Geosci. 16, 505–512 (2023).

[19] P. A. Cundall, O. D. L. Strack, The development of constitutive laws for soil using the distinct element method. Numer. Methods Geomech. 1, 289–317 (1979).

[20] J. K. Morgan, Effects of cohesion on the structural and mechanical evolution of fold and thrust belts and contractional wedges: Discrete element simulations. J. Geophys. Res. Solid Earth. 120, 3870–3896 (2015).

[21] A. Nicol, C. Mazengarb, F. Chanier, G. Rait, C. Uruski, L. Wallace, Tectonic evolution of the active Hikurangi subduction margin, New Zealand, since the Oligocene. Tectonics 26, TC4002 (2007).

[22] D. Bassett, G. Fujie, S. Kodaira, R. Arai, Y. Yamamoto, S. Henrys, D. Barker, A. Gase, H. van Avendonk, N. Bangs, H. Seebeck, B. Tozer, K. Jacobs, T. Luckie, D. Okaya, K. Mochizuki, Heterogeneous crustal structure of the Hikurangi Plateau revealed by SHIRE seismic data: Origin and implications for plate boundary tectonics. Geophys. Res. Lett. 50, e2023GL105674 (2023).

[23] A. C. Gase, N. L. Bangs, H. J. A. Van Avendonk, D. Bassett, S. Henrys, R. Arai, G. Fujie, P. M. Barnes, S. Kodaira, D. H. N. Barker, D. Okaya, Volcanic crustal structure of the western Hikurangi Plateau from marine seismic reflection imaging. Geosphere 20, 935–964 (2024).

[24] G. A. Eiby, Two New Zealand tsunamis. J. R. Soc. N. Z. 12, 338–351 (1982).

[25] D. I. Doser, T. H. Webb, Source parameters of large historical (1917–1961) earthquakes, North Island, New Zealand. Geophys. J. Int. 152, 795–832 (2003).

[26] L. M. Wallace, D. M. Saffer, P. M. Barnes, I. A. Pecher, K. E. Petronotis, L. J. LeVay, Expedition 372/375 Scientists, “Hikurangi subduction margin coring, logging, and observatories,” in *Proceedings of the International Ocean Discovery Program* (International Ocean Discovery Program, 2019).

[27] C. Chesley, S. Naif, K. Key, D. Bassett, Fluid-rich subducting topography generates anomalous forearc porosity. Nature 595, 255–260 (2021). 34234336 10.1038/s41586-021-03619-8

[28] R. L. Evans, Constraints on the large-scale porosity and permeability structure of young oceanic crust from velocity and resistivity data. Geophys. J. Int. 119, 869–879 (1994).

[29] S. Naif, K. Key, S. Constable, R. L. Evans, Water-rich bending faults at the Middle America Trench. Geochem. Geophys. Geosyst. 16, 2582–2597 (2015).

[30] A. C. Gase, N. L. Bangs, D. M. Saffer, S. Han, P. K. Miller, R. E. Bell, R. Arai, S. A. Henrys, S. Kodaira, R. Davy, L. Frahm, D. H. N. Barker, Subducting volcaniclastic-rich upper crust supplies fluids for shallow megathrust and slow slip. Sci. Adv. 9, eadh0150 (2023). 37585538 10.1126/sciadv.adh0150 PMC10431706

[31] C. Chang, M. D. Zoback, A. Khaksar, Empirical relations between rock strength and physical properties in sedimentary rocks. J. Petrol. Sci. Eng. 51, 223–237 (2006).

[32] H. Leah, Å. Fagereng, F. Meneghini, J. K. Morgan, H. M. Savage, M. Wang, R. Bell, M. J. Ikari, Mixed brittle and viscous strain localization in pelagic sediments seaward of the Hikurangi Margin, New Zealand. Tectonics 39, e2019TC005965 (2020).

[33] H. R. Shaddox, S. Y. Schwartz, Subducted seamount diverts shallow slow slip to the forearc of the northern Hikurangi subduction zone, New Zealand. Geology 47, 415–418 (2019).

[34] D. M. Saffer, H. J. Tobin, Hydrogeology and mechanics of subduction zone forearcs: Fluid flow and pore pressure. Annu. Rev. Earth Planet. Sci. 39, 157–186 (2011).

[35] T. Sun, D. Saffer, S. Ellis, Mechanical and hydrological effects of seamount subduction on megathrust stress and slip. Nat. Geosci. 13, 249–255 (2020).

[36] H. Leah, Å. Fagereng, I. Bastow, R. Bell, V. Lane, S. Henrys, K. Jacobs, B. Fry, The northern Hikurangi margin three-dimensional plate interface in New Zealand remains rough 100 km from the trench. Geology 50, 1256–1260 (2022).

[37] E. Van Rijsingen, F. Funiciello, F. Corbi, S. Lallemand, Rough subducting seafloor reduces interseismic coupling and mega-earthquake occurrence: Insights from analogue models. Geophys. Res. Lett. 46, 3124–3132 (2019).

[38] S. Lallemand, M. Peyret, E. van Rijsingen, D. Arcay, A. Heuret, Roughness characteristics of oceanic seafloor prior to subduction in relation to the seismogenic potential of subduction zones. Geochem. Geophys. Geosyst. 19, 2121–2146 (2018).

[39] A. C. Gase, N. L. Bangs, H. J. Van Avendonk, D. Bassett, S. A. Henrys, Hikurangi megathrust slip behavior influenced by lateral variability in sediment subduction. Geology 50, 1145–1149 (2022).

[40] R. M. Skarbek, A. W. Rempel, D. A. Schmidt, Geologic heterogeneity can produce aseismic slip transients. Geophys. Res. Lett. 39, L21306 (2012).

[41] E. Todd, S. Schwartz, C. Williams, A. Sheehan, K. Mochizuki, L. M. Wallace, A. F. Sheehan, S. C. Webb, C. A. Williams, J. Nakai, J. Yarce, B. Fry, S. Henrys, Y. Ito, Earthquakes and tremor linked to seamount subduction during shallow slow slip at the Hikurangi margin, New Zealand. J. Geophys. Res. Solid Earth 123, 6769–6783 (2018).

[42] D. H. Barker, S. Henrys, F. Caratori Tontini, P. M. Barnes, D. Bassett, E. Todd, L. Wallace, Geophysical constraints on the relationship between seamount subduction, slow slip, and tremor at the north Hikurangi subduction zone, New Zealand. Geophys. Res. Lett. 45, 12–804 (2018).

[43] R. Bell, C. Holden, W. Power, X. Wang, G. Downes, Hikurangi margin tsunami earthquake generated by slow seismic rupture over a subducted seamount. Earth Planet. Sci. Lett. 397, 1–9 (2014).

[44] J. K. Morgan, N. L. Bangs, Recognizing seamount-forearc collisions at accretionary margins: Insights from discrete numerical simulations. Geology 45, 635–638 (2017).

[45] R. M. Kurzawski, M. Stipp, A. R. Niemeijer, C. J. Spiers, J. H. Behrmann, Earthquake nucleation in weak subducted carbonates. Nat. Geosci. 9, 717–722 (2016).

[46] A. M. Eijsink, M. J. Ikari, Plate-rate frictional behavior of sediment inputs to the Hikurangi subduction margin: How does lithology control slow slip events? Geochem. Geophys. Geosyst. 23, e2022GC010369 (2022).

[47] C. Li, H. Yin, C. Wu, Y. Zhang, J. Zhang, Z. Wu, W. Wang, D. Jia, S. Guan, R. Ren, Calibration of the discrete element method and modelling of shortening experiments. Front. Earth Sci. 9, 636512 (2021).

[48] P. Wessel, W. H. Smith, A global, self-consistent, hierarchical, high-resolution shoreline database. J. Geophys. Res. Solid Earth 101, 8741–8743 (1996).

[49] C. Botter, N. Cardozo, S. Hardy, I. Lecomte, A. Escalona, From mechanical modeling to seismic imaging of faults: A synthetic workflow to study the impact of faults on seismic. Mar. Pet. Geol. 57, 187–207 (2014).

[50] H. Tilley, G. Moore, M. Underwood, F. Hernández-Molina, M. Yamashita, S. Kodaira, A. Nakanishi, Heterogeneous sediment input at the Nankai Trough subduction zone: Implications for shallow slow earthquake localization. Geochem. Geophys. Geosyst. 22, e2021GC009965 (2021).

[51] N. Bangs, S. Han, R. Bell, D. Barker, R. Arai, S. Kodaira, J. Edwards, H. Tobin, G. Moore, S. Henrys, D. Bassett, NZ3D seismic reflection data volume—Prestack Depth Migration (PSDM), Marine Geoscience Data System (MGDS) (2022); 10.26022/IEDA/331022.

[52] NZ3D data processing team, NZ3D seismic data processing report (Marine Geoscience Data System, 2022); https://marine-geo.org/tools/files/31023#documents.

