# Supplementary material for: Effects of incoming polygonal fault systems on subduction zone and slow slip behavior
Source: Sci Adv. 2025 Jul 4;11(27):eadu4227. doi: 10.1126/sciadv.adu4227 (PMC12227072; doi:10.1126/sciadv.adu4227)
Supplement: Supplementary file 1 — Figs. S1 to S8 Table S1 Legends for movies S1 to S7 [file sciadv.adu4227_sm.pdf]

Science Advances
AAAS

Supplementary Materials for

Effects of incoming polygonal fault systems on subduction zone and slow slip behavior

Maomao Wang *et al.*

Corresponding author: Maomao Wang, wangmm@hhu.edu.cn

Sci. Adv. **11**, eadu4227 (2025)
DOI: 10.1126/sciadv.adu4227

The PDF file includes:

Figs. S1 to S8
Table S1
Legends for movies S1 to S7

Other Supplementary Material for this manuscript includes the following:

Movies S1 to S7

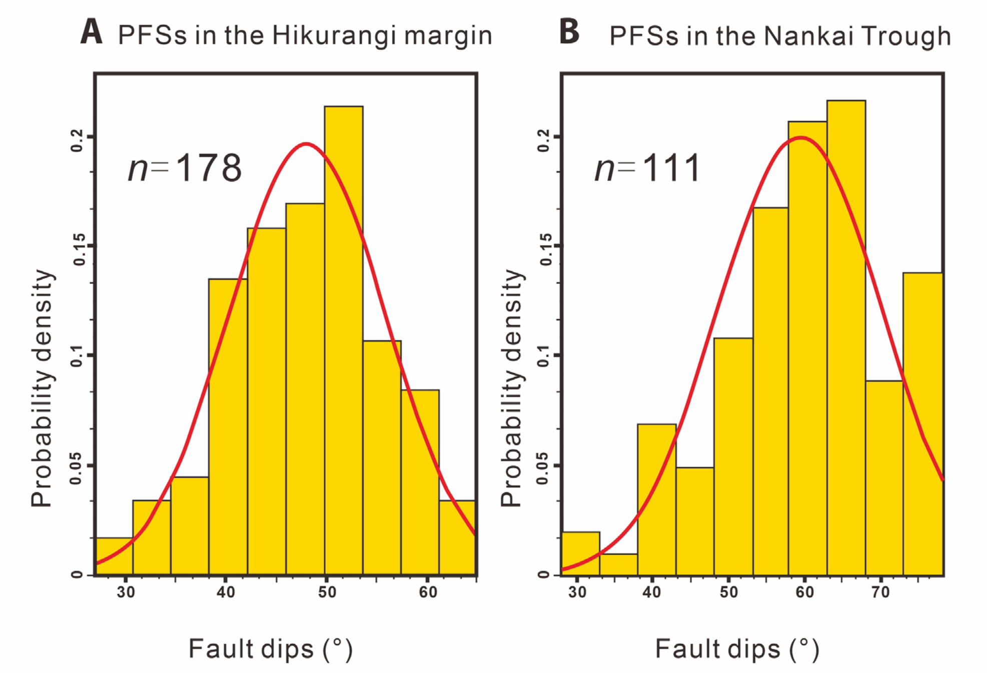

Fig. S1. Probability density functions of the PFSs fault dips derived from seismic reflection data in Hikurangi Margin (A) and Nankai Trough (B).

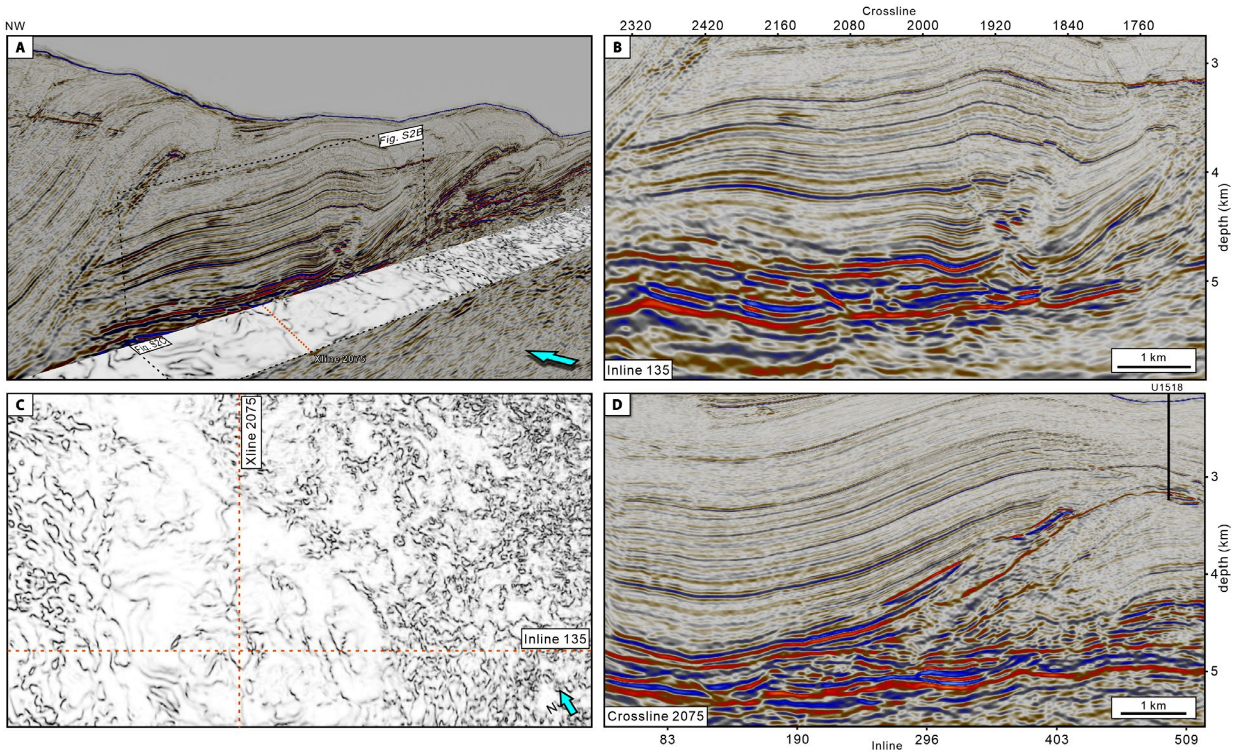

Fig. S2. Uninterpreted seismic profiles and coherent depth slice from the NZ3D reflection volume corresponding to Figure 4.

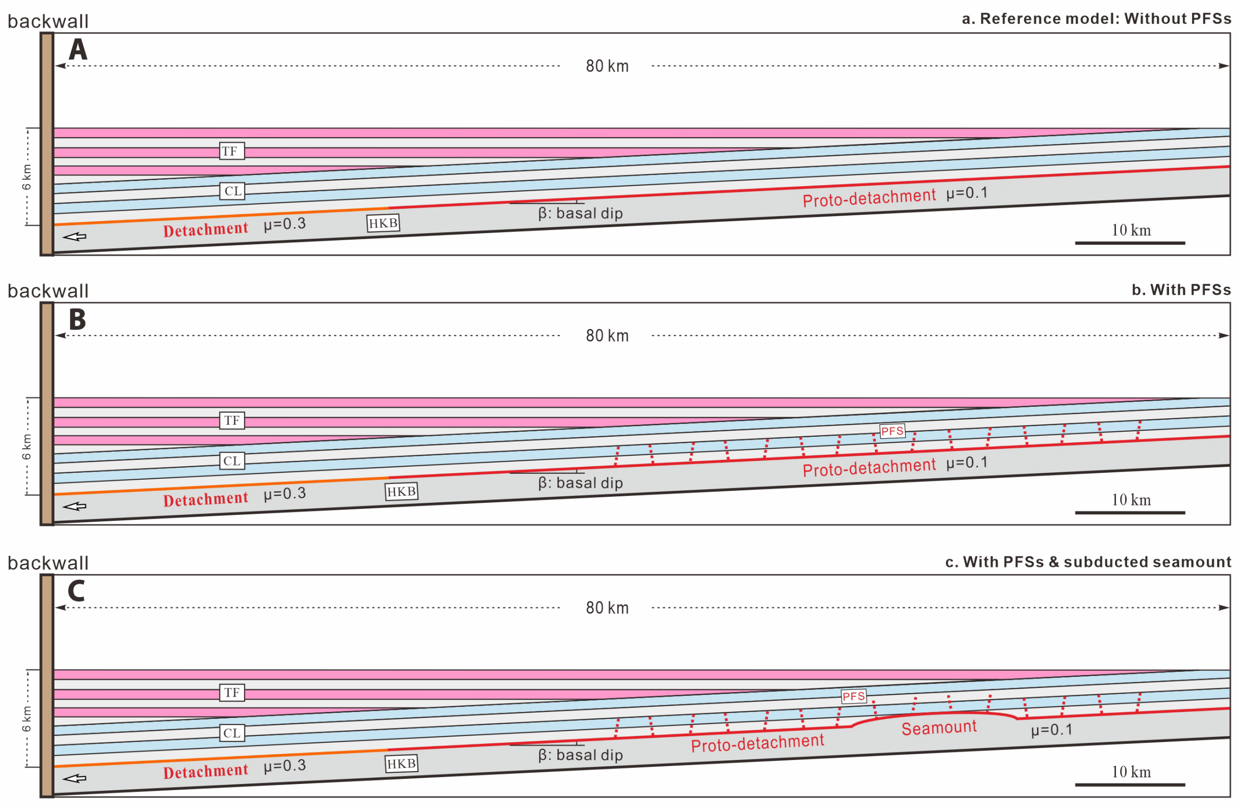

Fig. S3. Initial setup for the models without (A), with PFSs (B), seamount with PFSs (C) experiments. The seamount set as 15 km in width and 1 km in height.

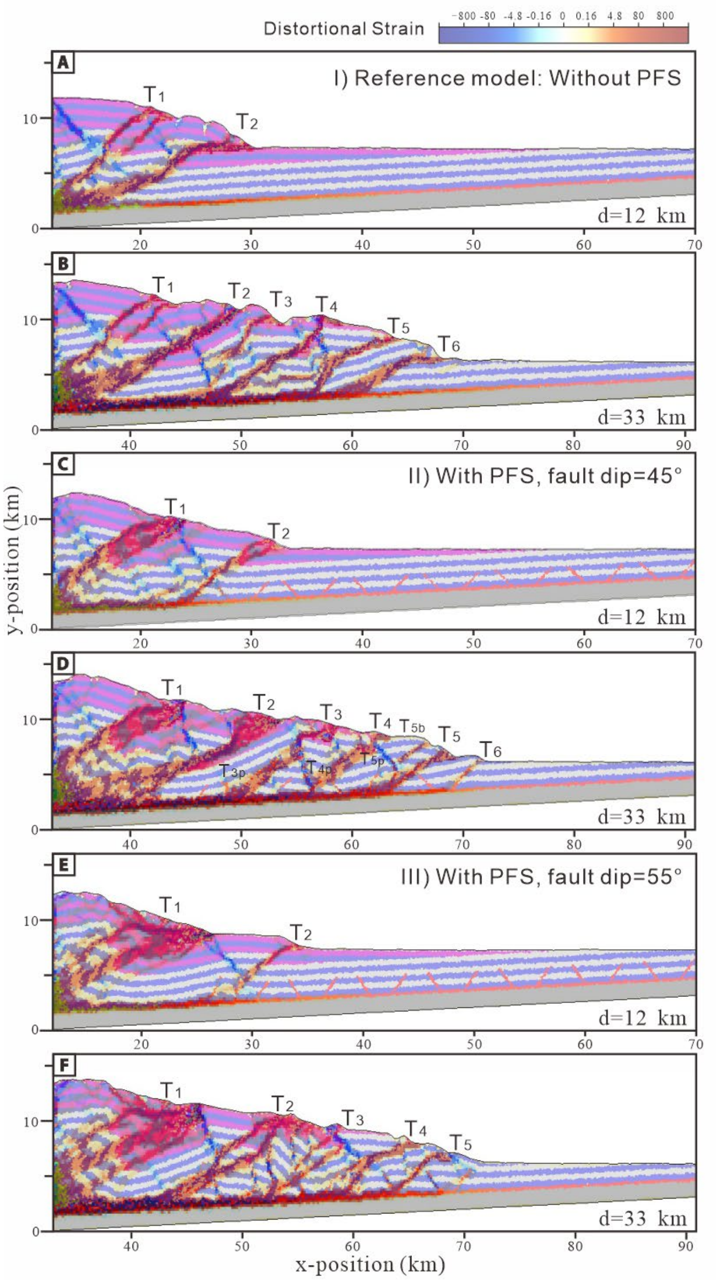

Fig. S4. Comparison of structural deformation of an accretionary wedge without (a-b) and with incoming PFSs fault dips of 45° (c-d) and fault dips of 55° (e-f), respectively, based on DEM at model displacement of 12 and 33 km. The red + blue and white + blue areas represent trench fill and pelagic sediment units, respectively, while the detachment is represented by green and red layers, corresponding to high friction coefficient (0.3) and low friction coefficient (0.1).

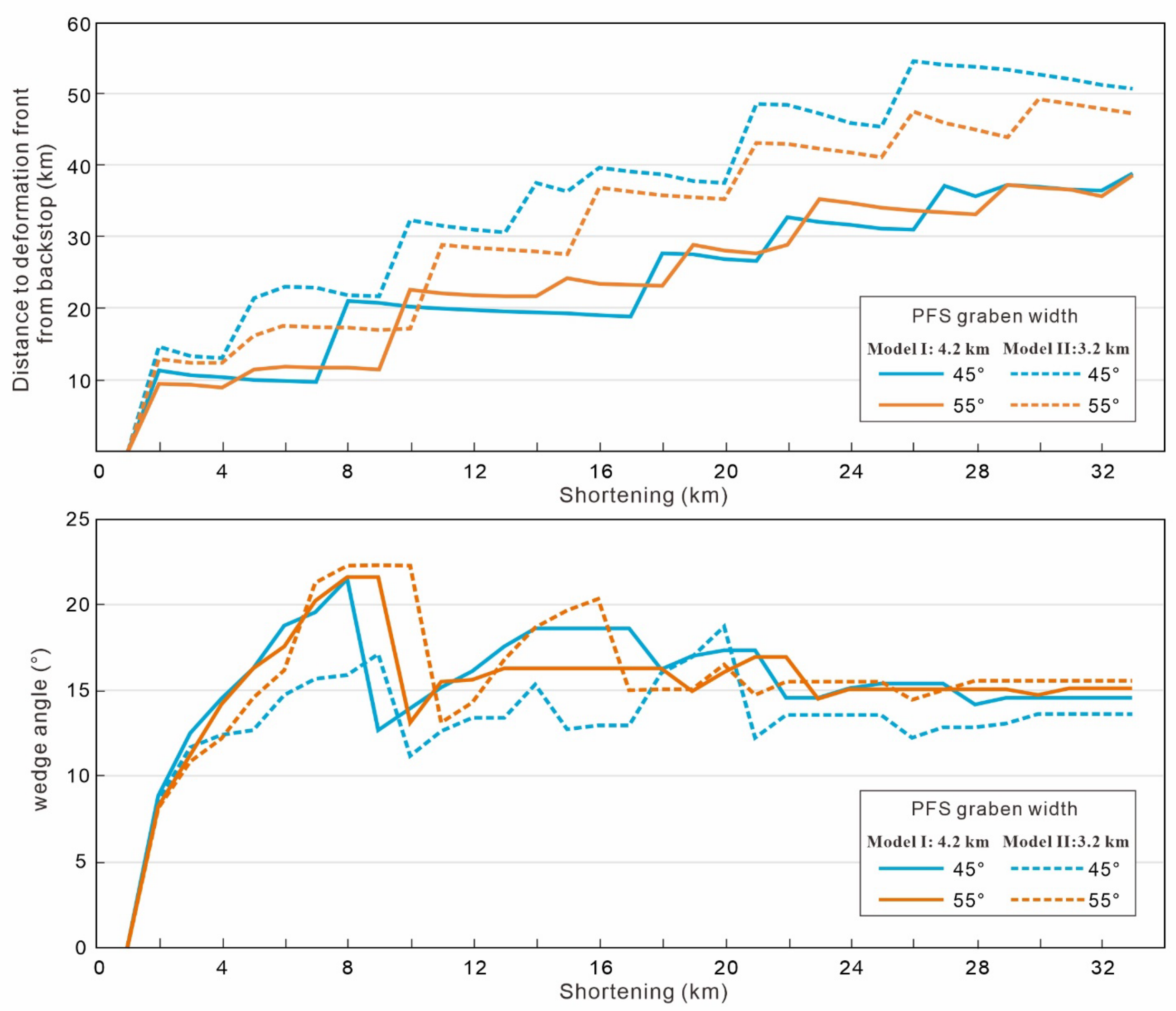

Fig. S5. Graphs showing a comparison of wedge width (distance from the deformation front to the backstop) versus mean wedge surface slopes in Models I and II for different PFSs graben spacings.

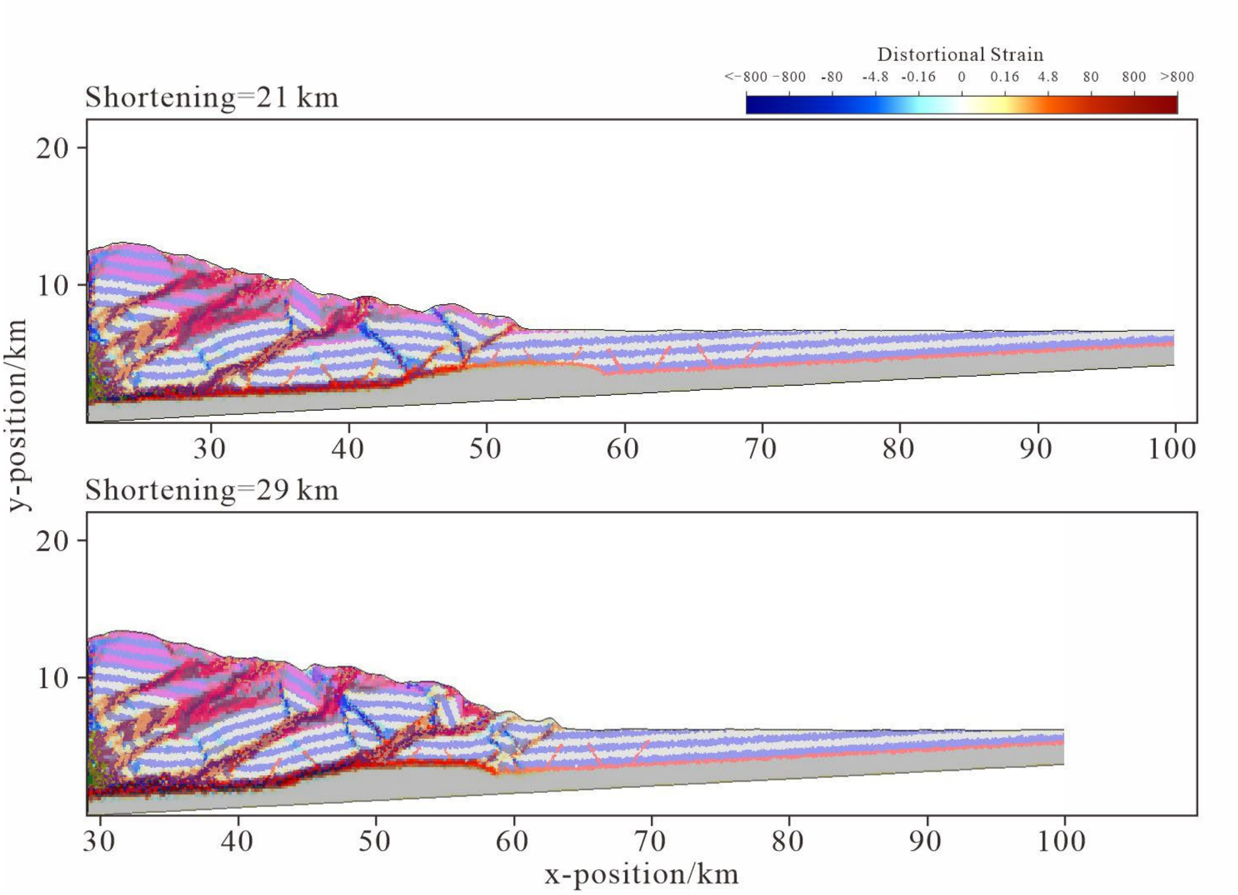

Fig. S6. 2D DEM Simulation results with PFSs and seamount model at 21 and 29km tectonic shortening, respectively. The fault dip of the PFSs was set to 55°, and the width of the seamount was 15 km with a height of 1 km.

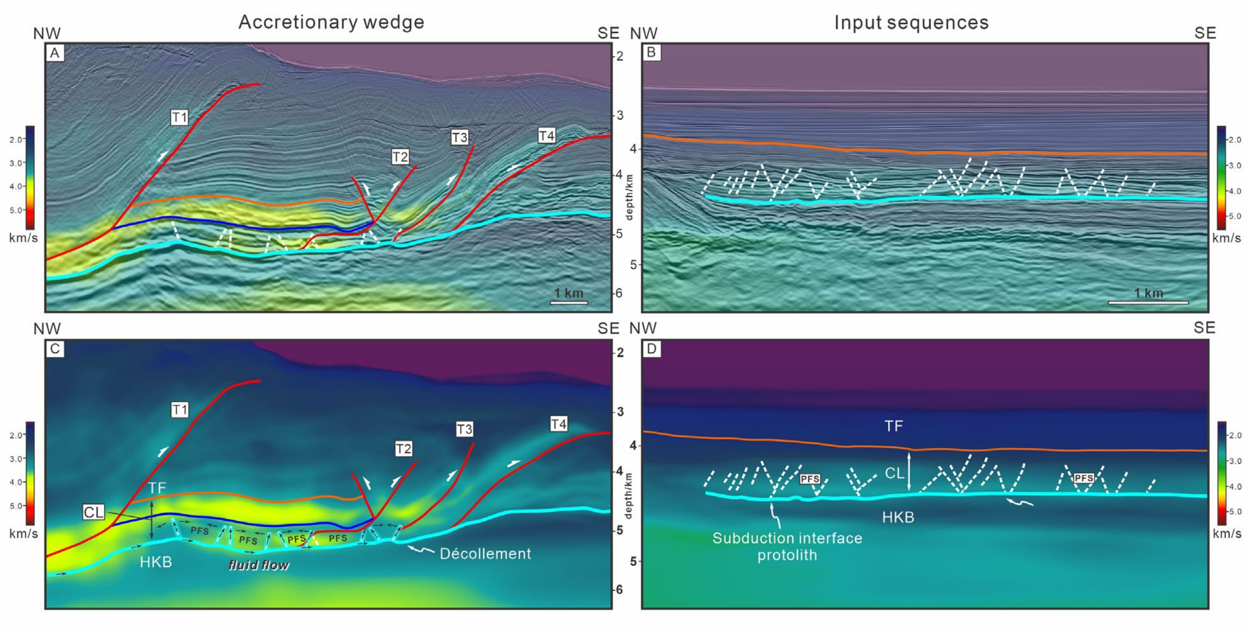

Fig. S7. **P-wave velocity model and seismic reflection profile derived from NZ3D in the Hikurangi accretionary wedge (A, C) and input sequences (B, D).**

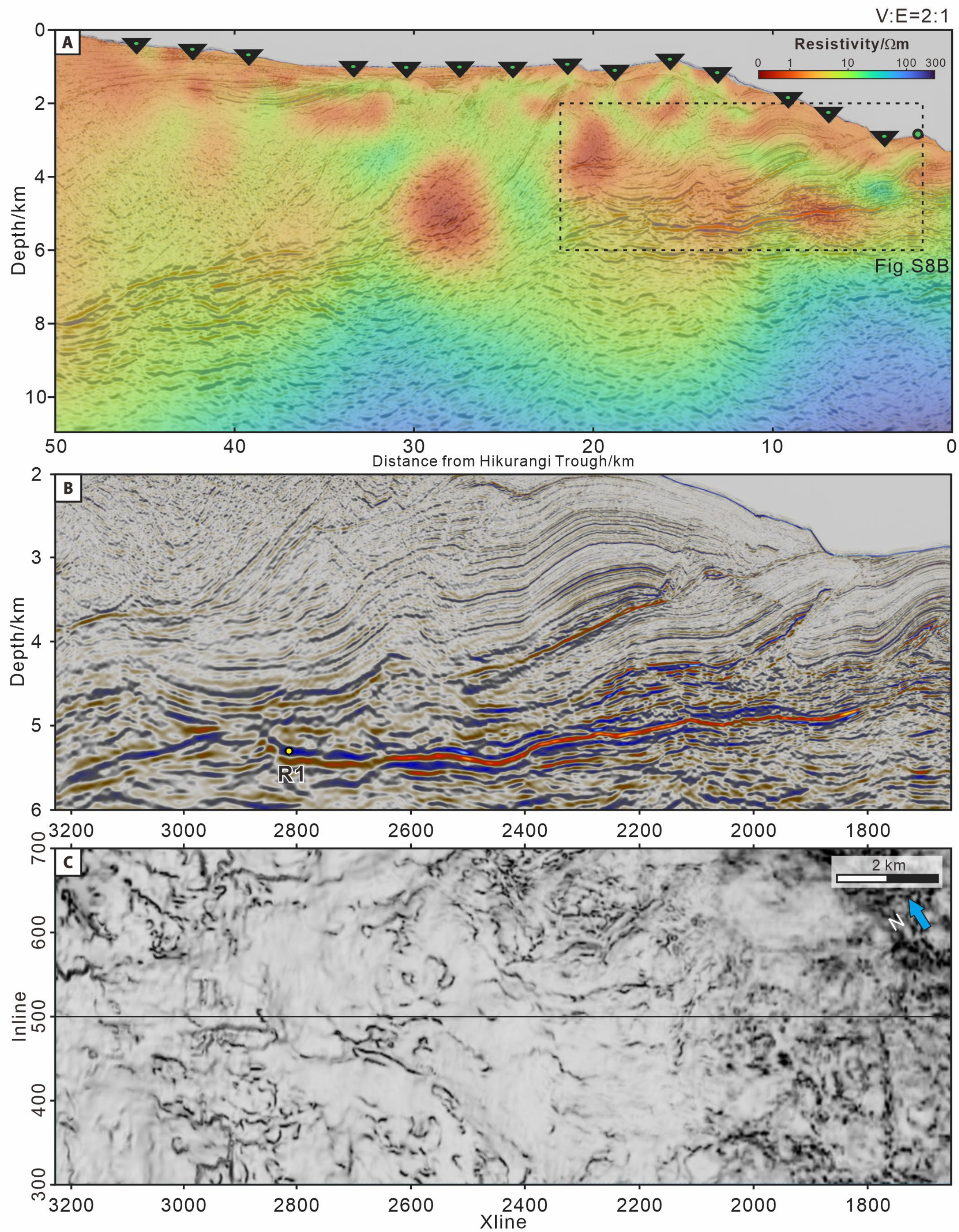

Fig. S8. Uninterpreted version of co-located seismic profile IL 500 from NZ3D overlain on resistivity. (B) Enlarged seismic reflection profile corresponding to the C3f, C4f region. (C) Seismic variance attribute map for the R1 reflection layer in Unit IV.

Table 1
Bulk Mechanical Properties Used for the DEM Simulations

	Particle properties [a]		Interparticle bond properties				Macro parameters [b]	
Units	Friction coefficient	Density (kg/m³)	Young's modulus (Pa)	Shear modulus (Pa)	Tensile strength (Pa)	Shear strength (Pa)	Cohesion C_o (MPa)	Internal friction angle (f) (°)
Trench-fill sediments (unit TF)	0.3	2,500	2.0E08	2.0E08	1.0E07	2.0E07	10.5	18.6
Pelagic carbonates (unit CL)	0.3	2,500	2.0E08	2.0E08	2.0E07	4.0E07	19.0	19.3
Detachment	0.3/0.1	2,200					1.2/1.6	20.0/13.0
Polygonal faults	0.1	2,200					1.6	13.0

[a] Radii: 60/80 m, shear modulus: 2.9E09 Pa, Poisson's ratio: 0.2, and wall velocity: 2 m/s. [b] Morgan (2015).

Table S1. Bulk mechanical properties used for the DEM experiments.

Legend for Supplementary Movie S1

Sequential evolution of the particles of the reference model (without PFSs) and three compressional models (with PFSs), superimposed on distortional strain field.

The fault dip of the PFS in three compressional experiments are set to 35°, 45°, 55°, respectively. The red + blue and white + blue areas represent trench fill and pelagic sediment units, respectively, while the detachment is represented by green and red layers, corresponding to high friction coefficient (0.3) and low friction coefficient (0.1).

Legend for Supplementary Movie S2

Sequential evolution of the particles of the reference model (without PFSs) and three compressional models (with PFSs), superimposed on distortional strain field.

The fault dip of the PFS in three compressional experiments are set to 55°, 65°, 75°, respectively. The red + blue and white + blue areas represent trench fill and pelagic sediment units, respectively, while the detachment is represented by green and red layers, corresponding to high friction coefficient (0.3) and low friction coefficient (0.1).

Legend for Supplementary Movie S3

Sequential evolution of the reference model (without PFSs) and three compressional models (with PFSs), superimposed on maximum shear stress (τ_{max}).

The fault dip of the PFSs in three compressional experiments are set to 35°, 45°, 55°, respectively.

Legend for Supplementary Movie S4

Sequential evolution of the reference model (without PFSs) and three compressional models (with PFSs), superimposed on maximum shear stress (τ_{max}).

The fault dip of the PFSs in three compressional experiments are set to 55°, 65°, 75°, respectively.

Legend for Supplementary Movie S5

Sequential evolution of the PFSs-bearing model, superimposed on distortional strain field.

The graben and horst in the PFSs is set as 3.2 km and 1.2 km. The red + blue and white + blue areas represent trench fill and pelagic sediment units, respectively, while the detachment is represented by green and red layers, corresponding to high friction coefficient (0.3) and low friction coefficient (0.1).

Legend for Supplementary Movie S6

Sequential evolution of the PFSs-bearing model, superimposed on maximum shear stress (τ_{max}).

The graben and horst in the PFSs is set as 3.2 km and 1.2 km,

Legend for Supplementary Movie S7

Sequential evolution of the model with PFSs and a seamount.

Upper: Sequential evolution of the model with PFSs and a seamount, superimposed on distortional strain field. Lower: Sequential evolution of corresponding distortional strain field. The fault dip of the PFSs was set to 55°, and the width of the seamount was 15 km with a height of 1 km. The red + blue and white + blue areas represent trench fill and pelagic sediment units,

respectively, while the detachment is represented by green and red layers, corresponding to high friction coefficient (0.3) and low friction coefficient (0.1).
